# Ferroptosis Inhibition Enhances Osteoblast Activity: The Role of Liproxstatin-1 and Coenzyme Q10

**DOI:** 10.3390/ijms262412059

**Published:** 2025-12-15

**Authors:** Alireza Valanezhad, Tetsurou Odatsu, Farzaneh Valanezhad, Shigeaki Abe, Ikuya Watanabe

**Affiliations:** 1 Department of Dental and Biomedical Materials Science, Graduate School of Biomedical Sciences, Nagasaki University, 1-7-1 Sakamoto, Nagasaki 852-8588, Japan; 2 Department of Applied Prosthodontics, Institute of Biomedical Sciences, Nagasaki University, 1-7-1, Sakamoto, Nagasaki 852-8588, Japan; 3 Department of Parasitology, Institute of Tropical Medicine (NEKKEN) and Graduate School of Biomedical Sciences, Nagasaki University, Nagasaki 852-8588, Japan

**Keywords:** Liproxstatin-1, Coenzyme Q10, ferroptosis, erastin, lipid peroxidation, bone formation

## Abstract

Ferroptosis, a form of regulated cell death triggered by lipid peroxidation, is implicated in various degenerative diseases and bone regeneration. In this study, we hypothesized that the ferroptosis inhibitors Liproxstatin-1 (Lip-1) and Coenzyme Q10 (CoQ10) play a dual role in protecting cells against ferroptotic damage and promoting osteogenic differentiation in MC3T3-E1 cells. Erastin-induced ferroptosis significantly reduced cell viability and increased lipid peroxidation, as evidenced by BODIPY™ 581/591 C11 staining. Both Lip-1 and CoQ10 decreased lipid peroxidation and restored cell viability, particularly at early treatment points. Post-treatment recovery experiments showed that both agents reversed erastin-induced damage, with Lip-1 having a stronger and more sustained effect. ALP activity assays on day 14 revealed dose-dependent increases with Lip-1 and moderate stimulation with CoQ10, indicating additional osteoinductive properties. Moreover, cell density affected sensitivity to lipid peroxidation, with higher cell densities providing protection through antioxidant pooling. These results highlight CoQ10 and Lip-1 as promising candidates for bone tissue engineering, as they offer protection against ferroptosis and promote osteoblast differentiation. Overall, this study emphasizes the therapeutic potential of ferroptosis modulators for bone regeneration.

## 1. Introduction

The development of osteogenesis, along with improvements in biomaterials and surgical techniques, has greatly enhanced the use of dental implants to restore function in partially and fully edentulous patients [1]. With global aging, bone-related complications, such as loosening of dental implants and hip fractures, are increasingly common and are largely associated with osteoporosis. Therefore, understanding the underlying mechanisms of cell death is essential for developing effective prevention and treatment strategies for bone degeneration [2]. Recent developments have expanded our understanding of cell death pathways beyond apoptosis and necrosis. It is now recognized that apoptosis is a fundamental form of programmed cell death, playing a crucial role in tissue homeostasis by removing damaged or excess cells and maintaining proper cell population balance [3,4]. Recent research has revealed that different forms of regulated necrosis, including necroptosis, ferroptosis, and pyroptosis, are governed by distinct molecular mechanisms and play critical roles in both normal physiology and disease progression [5]. According to Dixon et al. (2013) [6], ferroptosis is a distinct form of cell death that depends on iron and is characterized by extensive lipid peroxidation, which leads to membrane damage. Unlike apoptosis or traditional necrosis, ferroptosis occurs independently of caspase activity and ATP depletion, highlighting its unique molecular mechanism [6]. Ferroptosis occurs when the activities of glutathione peroxidase 4 (GPX4) and cystine/glutamate antiporter (system Xc^−^) are impaired, leading to reduced cysteine availability, decreased glutathione production, and consequently, insufficient detoxification of lipid peroxides, thereby promoting cell death [7,8]. Iron plays a vital role in several biological processes. However, both iron deficiency and overload can disturb the physiological balance, contributing to diseases such as anemia, cardiovascular disorders, liver disease, and cancer when iron homeostasis is impaired [9,10,11]. Ferroptosis is significantly driven by the Fenton reaction, in which ferrous ions (Fe^2+^) react with hydrogen peroxide to generate hydroxyl radicals. These radicals induce oxidative stress, a key driver of ferroptotic cell death, by promoting lipid peroxidation [12,13]. Excessive iron accumulation impairs osteoblast proliferation and differentiation by elevating oxidative stress through the generation of reactive oxygen species (ROS), ultimately triggering osteoblast apoptosis and contributing to osteoporosis the development of ferroptosis [14,15,16,17]. Targeting ferroptosis has proven effective in preventing osteoporosis by restoring osteoblast functions. Ferroptosis inhibits osteoblast activity and impairs bone formation, and its suppression contributes to improved skeletal integrity and bone health [18,19,20].

Erastin, a well-characterized ferroptosis inducer, triggers this iron-dependent form of cell death by inhibiting the cystine/glutamate antiporter (system Xc^−^), leading to glutathione exhaustion, uncontrolled lipid peroxidation, and eventual cellular collapse [6]. In this context, the cytotoxic effects of erastin were assessed in MC3T3-E1 pre-osteoblastic cells to elucidate its impact on cell viability and functional integrity [20,21]. Our previous investigation confirmed that ferrostatin-1, a potent ferroptosis suppressor, markedly enhanced both proliferation and osteogenic differentiation in MC3T3-E1 pre-osteoblast cells [20]. Moreover, Xiao et al. (2015) demonstrated that Townes transgenic sickle mice exhibit significantly diminished mRNA expression of key osteogenic markers, including alkaline phosphatase (ALP), suggesting that iron overload impairs osteoblast maturation [22]. Lip-1, a synthetically derived ferroptosis suppressor, functions as a potent lipid radical scavenger, effectively mitigating lipid peroxidation and safeguarding cells from oxidative injuries. Its efficacy has been validated in numerous disease models characterized by ferroptosis-driven pathology [23]. Although Lip-1 has been widely recognized for its potent ferroptosis-inhibitory properties and protective effects against oxidative stress in various disease models, its potential influence on osteogenesis remains unclear. To date, no comprehensive studies have directly evaluated whether Lip-1 modulates osteoblast proliferation, differentiation, or bone matrix formation. Therefore, further investigation is essential to determine whether its antioxidant and ferroptosis-suppressing capabilities contribute to bone regeneration or prevent bone loss under pathological conditions.

CoQ10 is a crucial lipid-soluble antioxidant that mitigates ferroptosis by neutralizing lipid peroxides in the cell membrane. Its reduced form effectively scavenges reactive lipid radicals, thereby inhibiting oxidative damage. Ferroptosis suppressor protein 1 (FSP1) sustains CoQ10 in its active, reduced state, enabling it to suppress ferroptosis independently of the glutathione peroxidase 4 (GPX4) pathway [24]. The intracellular transport of CoQ10 is orchestrated by carrier proteins such as STARD7, which facilitate its delivery to cellular compartments, where it mitigates ferroptosis [25]. Moreover, neuron-targeted CoQ10 formulations have demonstrated significant neuroprotective effects by alleviating oxidative stress and ferroptosis in neuronal tissues following conditions such as subarachnoid hemorrhage and epileptic seizures [26]. Additionally, CoQ10 exerts a pivotal influence on bone health by stimulating osteoblast proliferation and differentiation, mitigating oxidative stress, and suppressing bone resorption. Empirical evidence indicates that CoQ10 facilitates the formation of new bone tissue and attenuates osteoclast-driven bone degradation, positioning it as a compelling candidate for osteoporosis prevention and maintenance of skeletal integrity [27,28].

A novel fluorescent dye, 4,4-difluoro-5-(4-phenyl-1,3-butadienyl)-4-bora-3a,4a-diaza-s-indacene-3-undecanoic acid (commonly known as BODIPY™ 581/591 C11), has recently been developed for detecting lipid peroxidation [29]. This fluorophore comprises a boron dipyrromethene (BODIPY) difluoride backbone conjugated to a phenyl group via a diene linker and is appended with an undecanoic acid chain to facilitate its stable incorporation into lipid bilayers without cytotoxicity. When excited at 488 nm, BODIPY™ 581/591 C11 emits robust red fluorescence that peaks at approximately 595 nm [30]. Oxidation of the diene linker leads to cleavage of the phenyl moiety, triggering a fluorescence shift from red to a pronounced green signal at 520 nm. Notably, two principal oxidation derivatives have been identified, along with a third species generated specifically through peroxynitrite-mediated oxidation, which retains the phenyl group and exhibits peak fluorescence at 539 nm [31]. The landmark study by MacDonald et al. (2007) [32] was among the earliest to comprehensively characterize the chemical architecture, fluorescence behavior, and experimental utility of BODIPY™ 581/591 C11. Their work laid the foundation for a widely adopted protocol for lipid peroxidation assays and antioxidant evaluations [32].

In the present study, Lip-1, a well-known ferroptosis-inhibiting agent, and CoQ10, an osteogenic compound, were employed and compared. Therefore, the objective of this study was to investigate the effects of these compounds on erastin-induced ferroptotic cell death, as well as on the cell death rescue and osteogenic differentiation of MC3T3-E1 osteoblast cells.

## 2. Results

### 2.1. Erastin Induced Cell Death

**Figure 1:**
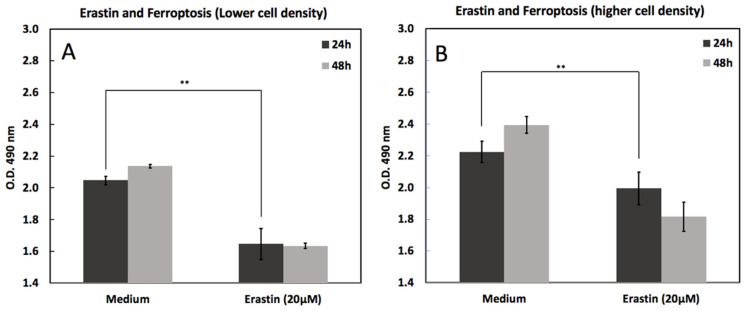
Erastin-induced cell death in MC3T3-E1 cells at low and high seeding densities. Cell viability was measured by MTS assay 24 h and 48 h after treatment with erastin (20 μM) compared with medium controls. (**A**) At lower cell density, erastin significantly reduced viability at both time points. (**B**) At higher cell density, cells similarly showed reduced viability following erastin treatment, with a more pronounced decline at 48 h. Data are presented as mean ± SD. ** indicates *p* < 0.05.

Figure 1 shows erastin-induced cell death under varying cell densities seeded at either 10,000 cells/mL (Figure 1A) or 20,000 cells/mL (Figure 1B) for 48 h and treated with 20 µM erastin or vehicle control (medium) for 24 and 48 h. Cell death was quantified and plotted as mean ± SEM. Erastin significantly reduced cell viability at both densities; however, the effect was more pronounced at lower seeding densities, suggesting density-dependent sensitivity to ferroptosis induction.

### 2.2. Optimum Lip-1 and CoQ10 Concentration

**Figure 2:**
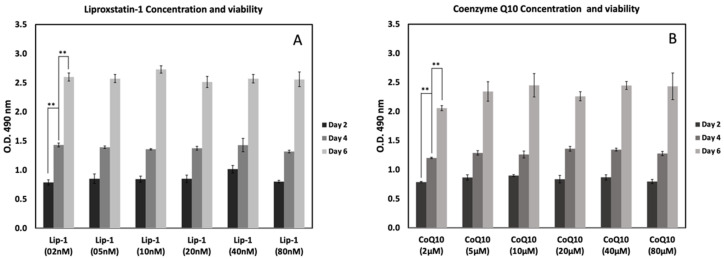
Effects of Liproxstatin-1 (Lip-1) and Coenzyme Q10 (CoQ10) concentrations on MC3T3-E1 cell viability at different time points. Cells were treated with increasing concentrations of Lip-1 (2–80 nM) (**A**) or CoQ10 (2–80 μM) (**B**), and viability was assessed by MTS assay on days 2, 4, and 6. Both compounds showed dose-dependent and time-dependent effects on cell metabolic activity, with higher readings observed at later time points. Data represent mean ± SD. ** indicates *p* < 0.05.

Figure 2 shows the effects of Lip-1 and CoQ10 concentrations on MC3T3-E1 cell viability after 2, 4, and 6 days of treatment. Treatment with different concentrations of Lip-1 (2–80 nM) demonstrated a dose-independent enhancement of cell viability over time (Figure 2A). Treatment with CoQ10 (2–80 μM) also promoted cell proliferation in a time-dependent manner in this study. The viability of MC3T3-E1 cells progressively increased from day 2 to day 6 with Lip-1 and CoQ10 treatment. In the Lip-1-treated groups, cell growth was relatively stable across concentrations, showing healthy proliferation by day 6 of treatment. Similarly, CoQ10-treated cells displayed a consistent increase in viability, particularly at higher doses (20–80 μM), on day 6 post-treatment. No cytotoxicity was observed at any of the tested concentrations.

### 2.3. Lip-1 and CoQ10 and Ferroptosis Inhibition

**Figure 3:**
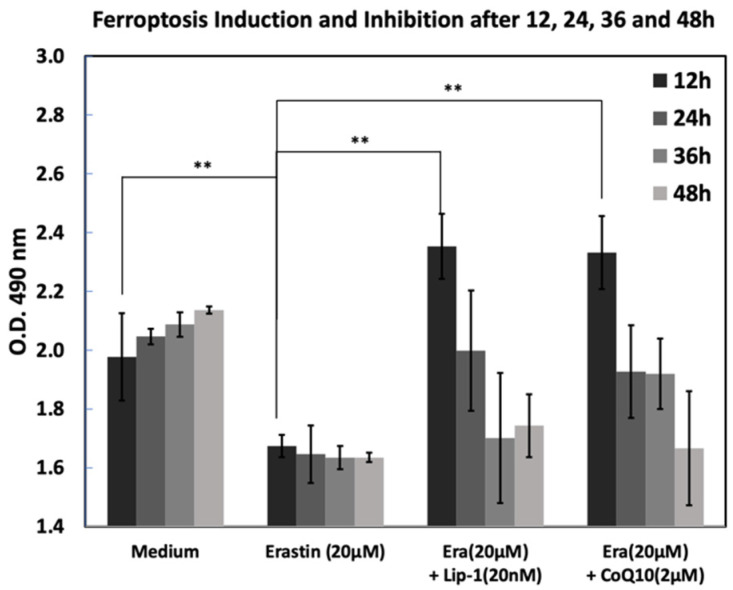
Time-course analysis of erastin-induced ferroptosis and its inhibition by Liproxstatin-1 and CoQ10 from 12 to 48 h. MC3T3-E1 cells were treated with medium control, erastin (20 μM), or erastin in combination with Lip-1 (20 nM) or CoQ10 (2 μM), and cell viability was measured by MTS assay at 12, 24, 36 and 48 h. Erastin progressively reduced cell viability over time, while co-treatment with Lip-1 or CoQ10 partially restored metabolic activity at earlier time points. Data are presented as mean ± SD. ** indicates *p* < 0.0.

To evaluate the dynamics of ferroptosis and its inhibition, cells were treated with erastin (20 µM) alone or in combination with Lip-1 (20 nM) or CoQ10 (2 µM), and cell viability was assessed at 12, 24, 36, and 48 h using absorbance at 490 nm (Figure 3). Cells in medium alone showed increased viability over time, whereas treatment with erastin significantly reduced optical density at all time points, confirming effective ferroptosis. Cells were treated with medium (Med), erastin (20 µM), or co-treated with either Lip-1 (20 nM) or CoQ10 (2 µM). Cell viability was measured at 12, 24, 36, and 48 h by measuring the absorbance at 490 nm. Erastin significantly reduced viability at all time points, whereas Lip-1 and CoQ10 effectively rescued cell viability, particularly 12 h after treatment. The protective effects of both inhibitors declined over time, although they remained partially effective at 48 h. Data are presented as the mean ± standard deviation (SD).

### 2.4. Cell Viability, Ferroptosis and Recovery

**Figure 4:**
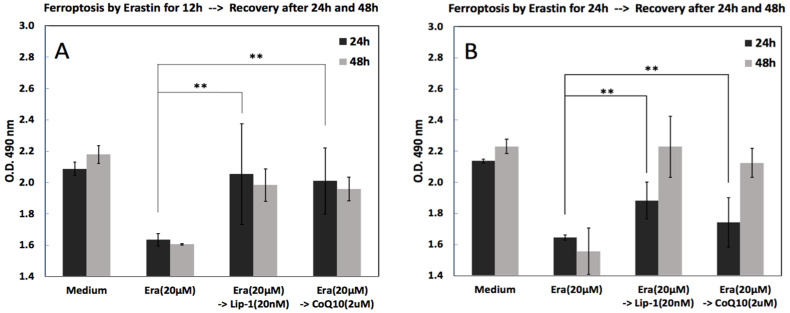
Post-treatment recovery from erastin-induced ferroptosis after early (12 h) or prolonged (24 h) exposure. MC3T3-E1 cells were first treated with erastin (20 μM) for either 12 h (**A**) or 24 h (**B**), followed by removal of erastin and rescue with Liproxstatin-1 (20 nM) or CoQ10 (2 μM). Cell viability was assessed 24 h and 48 h after rescue. Erastin alone significantly reduced cell viability, whereas subsequent treatment with Lip-1 or CoQ10 promoted partial recovery, with a more pronounced effect observed in the 12-h induction condition. Data represent mean ± SD. ** indicates *p* < 0.05.

To assess whether cell viability after ferroptosis induction could be recovered after treatment with Lip-1 and CoQ10, cells were first exposed to erastin (20 µM) for either 12 or 24 h, followed by a recovery phase with Lip-1 (20 nM) or CoQ10 (2 µM). As shown in Figure 4, erastin treatment decreased cell viability compared to the medium control in both experiments, confirming the successful induction of ferroptosis. Cells were exposed to erastin (20 µM) for 12 h (Figure 4A) or 24 h (Figure 4B), followed by treatment with Lip-1 (20 nM) or CoQ10 (2 µM) for an additional 24 or 48 h. Cell viability assays indicated that erastin reduced viability, whereas subsequent treatment with Lip-1 or CoQ10 significantly restored cell survival, especially after 12 and 24 h of stress followed by 48 h of recovery. Lip-1 demonstrated slightly higher efficacy than CoQ10 in restoring cell viability.

### 2.5. BODIPY™ 581/591 C11 and Hoechst^®^33342 Staining

#### 2.5.1. Ferroptosis or Lipid Peroxidation Imaging

**Figure 5:**
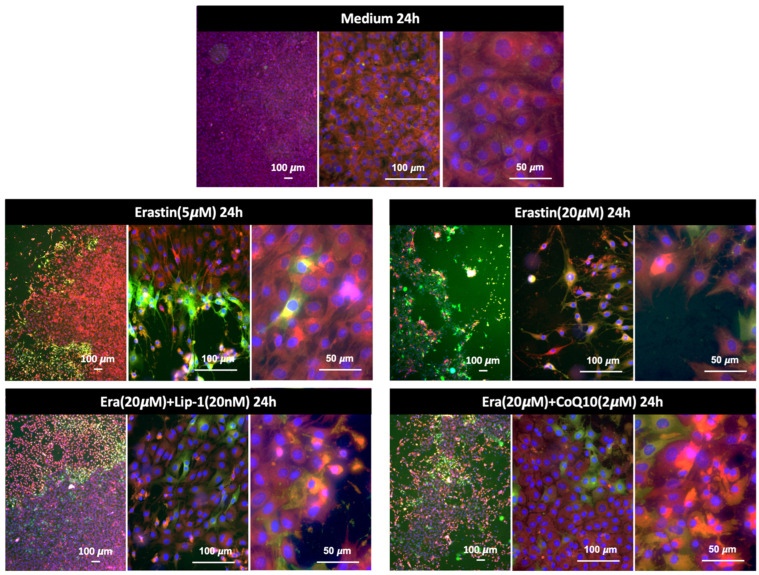
Lipid peroxidation in MC3T3-E1 cells detected by BODIPY™ 581/591 C11 staining after 24 h of treatment. Cells exposed to erastin at 5 μM or 20 μM showed a strong increase in lipid ROS, indicated by the shift from red to green fluorescence. Co-treatment with Liproxstatin-1 (20 nM) or CoQ10 (2 μM) markedly reduced lipid peroxidation, demonstrating the protective effects of ferroptosis inhibitors against erastin-induced oxidative damage. Representative images are shown at low, medium, and high magnification.

Figure 5 shows representative fluorescence images of MC3T3-E1 cells stained with BODIPY™ 581/591 C11 after treatment with erastin and ferroptosis inhibitors for 24 h. Assessment of lipid peroxidation and cell viability using BODIPY™ 581/591 C11 and Hoechst^®^33342 staining following erastin and rescue treatments. Cells were stained with BODIPY™ 581/591 C11, which shifts emission from red (reduced lipids) to green (oxidized lipids) upon peroxidation, and Hoechst^®^33342, which labels nuclei (blue) as an indicator of viability. Images were acquired at 4×, 20×, and 40× magnifications. In the medium control group, the cells displayed uniform red BODIPY staining and normal Hoechst-labeled nuclei, consistent with intact membrane and low basal oxidative stress. Erastin treatment (5 µM, 24 h) induced a noticeable increase in the green signal, indicating moderate lipid peroxidation. Hoechst^®^33342 staining revealed that many nuclei were still intact, but some cells exhibited nuclear condensation and shrinkage, suggesting partial cell death. At higher erastin concentrations (20 µM, 24 h), there was a dramatic shift from red to green fluorescence, accompanied by widespread loss of cell density. Hoechst^®^33342 staining revealed numerous fragmented or faintly stained nuclei, consistent with severe cell death. Numerous “washed-out” areas lacking nuclei were observed, indicating detachment of dead cells and suggesting that the majority of the cell population was lost under these conditions. Co-treatment with Liproxstatin-1 (20 nM) or Coenzyme Q10 (2 µM) markedly preserved cell density. BODIPY staining in these groups showed reduced green and maintenance of red signals, while Hoechst^®^33342 staining revealed mostly intact nuclei. In both cases, only a minority of cells displayed nuclear changes consistent with cell death, suggesting strong protective effects against erastin-induced ferroptosis. Scale bars: 100 µm (4×, 20×) and 50 µm (40×).

#### 2.5.2. Quantitative Lipid Peroxidation

**Figure 6:**
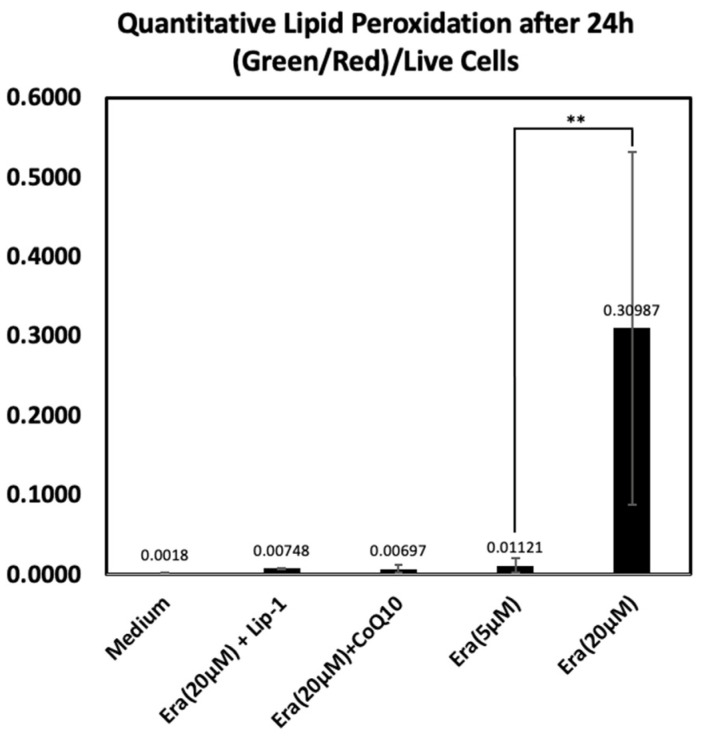
Quantitative lipid peroxidation analysis based on C11-BODIPY oxidation after 24 h. Green (oxidized) and red (reduced) fluorescence intensities were measured and normalized to the number of Hoechst-positive live cells using ImageJ, a web-based implementation (http://ij.imjoy.io) provided by the National Institutes of Health (NIH, Bethesda, MD, USA). Cells exposed to 20 µM erastin displayed a significant increase in lipid peroxidation, whereas Liproxstatin-1 and CoQ10 co-treatments effectively reduced oxidative lipid damage. Lower erastin concentration (5 µM) resulted in only minimal oxidation. Data represent mean ± SD. ** indicates *p* < 0.05.

Figure 6 shows a quantitative analysis of lipid peroxidation using the C11-BODIPY demonstrated a marked increase in oxidative lipid damage following exposure to erastin. Cells treated with 20 µM erastin showed a substantial elevation in the normalized green/red fluorescence ratio compared with all other treatment groups, indicating pronounced lipid peroxidation. In contrast, co-treatment with Liproxstatin-1 or CoQ10 markedly suppressed erastin-induced lipid oxidation, resulting in values comparable to the medium control. A mild increase was observed in the 5 µM erastin group but remained far lower than the 20 µM erastin condition. These results confirm that high-dose erastin triggers significant ferroptosis-associated lipid peroxidation and that this effect is efficiently attenuated by ferroptosis inhibitors.

#### 2.5.3. Ferroptosis or Lipid Peroxidation and Cell Density

**Figure 7:**
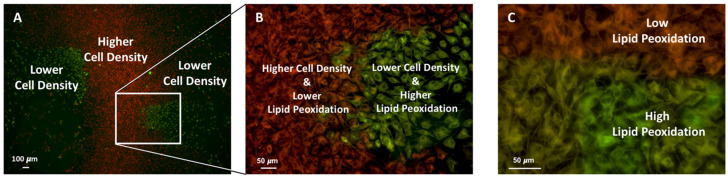
Differential lipid peroxidation in regions of low and high cell density following erastin treatment. MC3T3-E1 cells were exposed to 20 μM erastin for 12 h and stained with BODIPY™ 581/591 C11 to visualize lipid ROS levels. Low-density areas exhibited strong green fluorescence, indicating higher lipid peroxidation, whereas high-density areas showed predominantly red fluorescence, reflecting reduced oxidative stress (**A**,**B**). Magnified views highlight the inverse relationship between cell density and lipid peroxidation (**C**).

Figure 7 shows BODIPY™ 581/591 C11 staining to assess lipid peroxidation and ferroptosis in MC3T3-E1 cells treated with 20 μM erastin for 12 h. Figure 7A shows a low-magnification image of the two regions with red to orange and green-stained cells. Figure 7B shows, at a higher magnification, an area with a higher cell density, indicating low lipid peroxidation (orange fluorescence), and an area with a lower cell density and high lipid peroxidation (green fluorescence). Figure 7C shows a higher magnification of the boundary area, revealing a clear transition between low (orange) and high (green) lipid peroxidation.

### 2.6. Differentiation: ALP Activity

**Figure 8:**
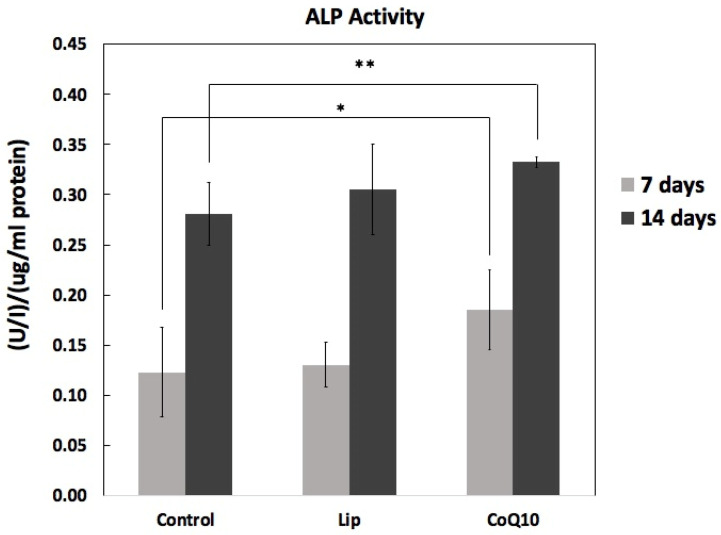
Effect of Liproxstatin-1 and Coenzyme Q10 on ALP activity on days 7 and 14. ALP activity was measured in the cell lysates and normalized to protein content. The results are shown as mean ± SD (*n* = 5). Both Lip and CoQ10 increased ALP activity compared to that in the control, with CoQ10 showing a more pronounced effect. Statistical analysis: * *p* < 0.05, ** *p* < 0.01 versus control.

Figure 8 shows alkaline phosphatase (ALP) activity, measured on days 7 and 14 under different treatment conditions. At day 7, ALP activity was modest in the control group (0.123 ± 0.044 U/L/µg protein) and slightly increased in the Lip-treated group (0.131 ± 0.023 U/L/µg protein), but a more pronounced increase was observed with CoQ10 treatment (0.173 ± 0.031 U/L/µg protein). On day 14, all groups showed higher ALP activity than on day 7, reflecting enhanced osteoblast differentiation with culture duration. Notably, CoQ10 (0.332 ± 0.006 U/L/µg protein) exhibited the greatest enhancement in ALP activity compared to the control (0.281 ± 0.031 U/L/µg protein), while Lip treatment (0.305 ± 0.045 U/L/µg protein) also promoted ALP activity, but to a lesser extent. Statistical analysis revealed that CoQ10 treatment significantly increased ALP activity compared to the control on days 7 (*p* < 0.05) and 14 (*p* < 0.01).

## 3. Discussion

### 3.1. Cell Density–Dependent Modulation of Ferroptosis Sensitivity

Our findings demonstrate that cell density markedly influences the susceptibility of MC3T3-E1 osteoblasts to erastin-induced ferroptosis. At low seeding density (10,000 cells/mL), erastin significantly reduced viability, whereas cells at higher density (20,000 cells/mL) exhibited partial resistance. Also, our group has previously reported the effect of cell density on cell viability during ferroptosis [20]. This density-dependent phenotype was further supported by BODIPY™ 581/591 C11 staining, which revealed pronounced lipid peroxidation (green fluorescence) in sparse regions and reduced peroxidation (red fluorescence) in densely packed areas. High-magnification imaging confirmed sharp spatial boundaries separating ferroptosis-sensitive and ferroptosis-resistant zones.

These observations align with prior reports demonstrating that cell density regulates ferroptosis sensitivity. For example, Panzilius et al. showed that low-density cultures accumulate PUFA-rich triacylglycerols, creating a pro-ferroptotic lipid landscape and enhancing vulnerability to lipid peroxidation [33]. In contrast, high-density conditions may promote adaptive metabolic or antioxidant mechanisms—such as increased glutathione content, altered PUFA utilization, or protective paracrine signaling—that attenuate lipid ROS accumulation. Together, these results indicate that cell density constitutes an important microenvironmental variable influencing ferroptosis outcomes and should be carefully controlled in experimental design.

### 3.2. Ferroptosis Inhibition and Recovery of Osteoblast Function

Erastin exposure induced a time-dependent decrease in MC3T3-E1 viability, confirming robust activation of ferroptosis. Co-treatment with Liproxstatin-1 or CoQ10 attenuated this decline at early time points, although their efficacy diminished after prolonged exposure, suggesting limited long-term protective capacity or cellular adaptation to sustained oxidative stress. Recovery experiments demonstrated that osteoblasts retained partial capacity for functional rescue when Lip-1 or CoQ10 was administered after erastin exposure, with Lip-1 generally producing a slightly greater restorative effect.

Importantly, the mechanisms by which Liproxstatin-1 and CoQ10 suppress ferroptosis are distinct and not attributable solely to general antioxidative activity. Liproxstatin-1 functions predominantly as a radical-trapping antioxidant (RTA) that directly intercepts lipid peroxyl radicals, thereby blocking lipid peroxidation chain reactions. This activity occurs independently of upstream metabolic pathways. In contrast, CoQ10 engages the FSP1–CoQ10 lipid redox pathway [34,35], representing a GPX4-independent ferroptosis-suppressing mechanism. Through FSP1-mediated reduction to ubiquinol, CoQ10 prevents membrane lipid oxidation at the plasma membrane. Additionally, CoQ10 supports mitochondrial function and bioenergetics, which may further contribute to osteoblast recovery and differentiation. These mechanistic distinctions emphasize that the two inhibitors act through complementary, biologically specific ferroptosis-regulatory pathways.

### 3.3. Implications for Osteogenic Differentiation

Both Liproxstatin-1 and CoQ10 enhanced ALP activity, indicating promotion of osteogenic differentiation. CoQ10 exerted a particularly strong effect, suggesting benefits beyond ferroptosis suppression, potentially through mitochondrial enhancement and improved oxidative balance. Lip-1 produced moderate but consistent increases in ALP activity, consistent with its capacity to limit lipid peroxidation and maintain cellular integrity during differentiation.

Collectively, these results highlight that ferroptosis regulation is closely linked to osteoblast functional maturation. By reducing lipid peroxidation and preserving intracellular homeostasis, ferroptosis inhibitors—particularly CoQ10—may support bone regeneration under oxidative stress conditions. These findings provide a mechanistic basis for further investigation of ferroptosis-targeting strategies in bone biology and regenerative medicine.

## 4. Materials and Methods

### 4.1. Materials

In this study, the MC3T3-E1 osteoblast cell line (Riken Cell Bank, Tokyo, Japan) was used to evaluate the cellular responses. Cells were cultured in MEM Alpha Medium (GIBCO, Invitrogen™, Carlsbad, NY, USA) supplemented with fetal bovine serum (FBS; GIBCO) and penicillin-streptomycin-glutamine (GIBCO). Cell viability was assessed using the MTS assay (CellTiter 96 Aqueous One Solution; Promega, Madison, WI, USA). For osteogenic differentiation, the medium was supplemented with ascorbic acid, β-glycerophosphate, and dexamethasone (all from Sigma-Aldrich, St. Louis, MO, USA). Phosphate-buffered saline (PBS; GIBCO) was used to wash the culture wells. Alkaline phosphatase (ALP) activity was measured using a commercial kit (Takara Bio, Shiga, Japan). Ferroptosis was induced using erastin (Sigma-Aldrich), 20 nM Lip-1, and 2 µM CoQ10 (Sigma-Aldrich). BODIPY™ 581/591 C11 (Invitrogen, Thermo Fisher Scientific, Waltham, MA, USA) was employed as a fluorescent probe to monitor lipid peroxidation, while Hoechst^®^33342 (Thermo Fisher Scientific) was used for nuclear staining to distinguish viable from dead cells during live-cell imaging.

### 4.2. Cell Viability

#### 4.2.1. Erastin Induced Cell Death

MC3T3-E1 cells were seeded in 96-well plates at two different densities: 10,000 and 20,000 cells/mL. After cell attachment for 48 h, the cells were treated with 20 µM erastin (ferroptosis inducer) for 24 and 48 h. The Control wells received medium without erastin. Cell viability was measured using the MTS CellTiter 96^®^ AQueous One Solution Cell Proliferation Assay (Promega, Madison, WI, USA), which relies on the bioreduction of MTS tetrazolium into a formazan product by metabolically active cells. At each time point, 20 µL of MTS reagent was added directly to each well containing 100 µL of medium and incubated at 37 °C for 3 h. Absorbance was measured at 490 nm using a microplate reader. Absorbance is directly proportional to the number of viable cells. Each condition was performed in triplicate, and the results are expressed as mean ± standard deviation (SD).

#### 4.2.2. Optimum Lip-1 and CoQ10 Concentration

MC3T3-E1 pre-osteoblast cells were seeded in 96-well plates at a density of 10,000 cells/well in 100 µL of complete α-MEM. The cells were incubated overnight at 37 °C in a humidified atmosphere with 5% CO_2_ for attachment. The following day, the cells were treated with varying concentrations of Lip-1 (2, 5, 10, 20, 40, and 80 nM) or CoQ10 (2, 5, 10, 20, 40, and 80 µM). The treatment medium was replaced every 48 h to maintain compound stability. Cell viability was assessed on days 2, 4, and 6 using the MTS assay (CellTiter 96^®^ AQueous One Solution; Promega, Madison, WI, USA). At each time point, 20 µL of MTS reagent was added directly to each well containing 100 µL of culture medium. The plates were incubated for 2 h at 37 °C, protected from light, and the absorbance was recorded at 490 nm using a microplate reader. All assays were performed in triplicate, and the results are expressed as mean ± SD.

#### 4.2.3. Lip-1 and CoQ10 and Ferroptosis Inhibition

MC3T3-E1 pre-osteoblast cells were cultured in complete α-MEM medium at 37 °C in a humidified atmosphere containing 5% CO_2_. Cells were seeded in 96-well plates at a density of 10,000 cells/well and allowed to adhere for 48 h. Ferroptosis was induced by treating the cells with erastin at a final concentration of 20 µM. To evaluate the protective effects against ferroptosis, the cells were co-treated with Lip-1 (20 nM) or CoQ10 (2 µM). The medium-only group served as a negative control. Cell viability was assessed using the CellTiter 96^®^ AQueous One Solution Cell Proliferation Assay (MTS assay) (Promega, Madison, WI, USA). At 12, 24, 36, and 48 h post-treatment, 20 µL of MTS reagent was added to each well containing 100 µL of the culture medium. The plates were incubated for 3 h at 37 °C. Absorbance was measured at 495 nm using a microplate reader to evaluate viable cell populations

#### 4.2.4. Cell Viability, Ferroptosis and Recovery

MC3T3-E1 cells were seeded at a density of 10,000 cells/mL in 96-well plates and allowed to adhere overnight in standard growth medium (α-MEM supplemented with 10% FBS and 1% penicillin-streptomycin) at 37 °C with 5% CO_2_. Cells were treated with erastin (20 μM) to induce ferroptosis for 12 or 24 h. A medium-only group was used as a control and was not exposed to erastin. After the ferroptosis induction period, the erastin-containing medium was removed, and cells were washed twice with PBS to eliminate residual erastin. Fresh growth medium containing no supplements (control recovery), Lip-1 (20 nM), or CoQ10 (2 μM) was added to the respective wells. The recovery phase continued for 24 or 48 h. Cell viability was evaluated using the MTS assay (CellTiter 96^®^ AQueous One Solution, Promega Madison, WI, USA) following the manufacturer’s protocol.

### 4.3. BODIPY™ 581/591 C11: Ferroptosis or Lipid Peroxidation Imaging

Boron dipyrromethane difluoride BODIPY™ 581/591 C11 is a fluorescent fatty acid analog commonly used to detect lipid peroxidation by measuring oxidized phospholipids within cells. Upon oxidation of its polyunsaturated butadienyl moiety, BODIPY™ 581/591 C11 undergoes a spectral shift in fluorescence emission from red to green, making it a sensitive ratiometric probe for lipid ROS detection [36].

Lip-1 (20 nM) and CoQ10 (2 μM) were used as ferroptosis inhibitors. BODIPY™ 581/591 C11 (Thermo Fisher, Waltham, MA, USA) was used to evaluate lipid peroxidation in living cells. This ratiometric fluorescent dye localizes to cellular membranes and exhibits a shift in fluorescence emission from red (~590 nm) to green (~510 nm) upon oxidation, making it a reliable sensor for detecting lipid ROS and oxidative stress during ferroptosis. Cells treated with erastin to induce ferroptosis were incubated with BODIPY™ 581/591 C11, and an All-in-one microscopic imaging system (BZ-X800; KEYENCE, Osaka, Japan) was used to image the samples.

To show the effect of cell density on the lipid peroxidation MC3T3-E1 cells were prepared at a concentration of 15,000 cells/mL in complete culture medium. Initially, 100 µL of the cell suspension was gently added to the center of a glass-bottom dish to allow the cells to preferentially attach to the central area. The dish was incubated at 37 °C in a humidified incubator with 5% CO_2_ for 10 min without disturbance, enabling partial cell attachment and creating a high-density region in the center. Subsequently, 2 mL of pre-warmed complete medium was carefully added to the dish to evenly cover the remaining surface. This procedure resulted in the formation of a distinct high-density area at the center, surrounded by a low-density cell area toward the periphery. The cells were then incubated under standard culture conditions for further experiments.

### 4.4. Hoechst^®^33342: Live-Cell Imaging

Cells were seeded in four-well chamber slides and allowed to adhere for 24 h. Subsequently, the cells were treated with the indicated conditions for 24 h, followed by staining under BODIPY™ 581/591 C11. After lipid peroxidation staining, cells were counterstained with Hoechst^®^33342 (Thermo Fisher Scientific) to visualize nuclei and evaluate cell viability. A 10 mg/mL stock solution of Hoechst^®^33342 was prepared in sterile distilled water. For live-cell imaging, the dye was diluted directly into pre-warmed phenol red–free medium to a final concentration of 1 µg/mL. Following treatment with erastin and/or rescue compounds, Hoechst^®^33342 was added to the culture medium for the last 10 min of incubation at 37 °C. The cells were protected from light during the staining. After staining, live cells were imaged directly in the medium to avoid detachment of dead cells.

### 4.5. Quantitative Lipid Peroxidation

Lipid peroxidation was quantified using the C11-BODIPY 581/591 fluorescent probe, which undergoes a spectral shift from red to green upon oxidation. Following treatment, cells were incubated with C11-BODIPY and washed with PBS, and imaged immediately using a fluorescence microscope. To determine cell viability and normalize fluorescence measurements to cell number, Hoechst 33342 to label nuclei of live cells. Hoechst-positive nuclei were subsequently counted from the blue fluorescence channel and used as a measure of live cell number. Differences in lipid peroxidation were assessed by quantifying oxidized (green) and reduced (red) C11-BODIPY fluorescence. Red and green fluorescence intensities were extracted from their respective channels and quantified using ImageJ, a web-based implementation (http://ij.imjoy.io) provided by the National Institutes of Health (NIH, Bethesda, MD, USA. To account for variation in cell density or viability between conditions, green and red fluorescence intensities were normalized to the number of Hoechst-positive nuclei. The oxidation status of the probe was expressed as the ratio of oxidized to reduced fluorescence (green/red), or as fluorescence intensity per live cell, depending on the analysis. Identical thresholding and particle-analysis parameters were applied across all samples to ensure consistent quantification.

### 4.6. Differentiation: ALP Activity

MC3T3-E1 pre-osteoblasts were seeded in 96-well plates and cultured in osteogenic differentiation medium supplemented with various concentrations of Lip-1 (5, 10, and 20 nM) and CoQ10 (0.5, 1, and 2 µM). The medium-only group served as a negative control. The cells were maintained at 37 °C in a humidified incubator with 5% CO_2_ for 14 days. The differentiation medium was refreshed every three days. At the end of the incubation period, ALP activity was evaluated using a TRACP and ALP assay kit (Takara Bio, Shiga, Japan). The cells were washed with PBS, and 50 µL of extraction solution was added to lyse the cells. Subsequently, the ALP buffer solution mixed with p-nitrophenyl phosphate (pNPP) was added as a substrate solution and incubated at 37 °C for 30 min to allow color development. The reaction was stopped by adding a stop solution, and the optical density was measured at 405 nm using a microplate reader to quantify ALP activity as an indicator of osteogenic differentiation.

### 4.7. Statistical Analysis

Data are expressed as mean ± standard deviation. Parametric analyses were performed using one-way ANOVA, followed by *t*-test comparisons. Statistical significance was set at *p*-value < 0.05.

## 5. Conclusions

This study highlights ferroptosis as a critical, yet underexplored, determinant of osteoblast viability and differentiation. By demonstrating that targeted inhibition of lipid peroxidation can both preserve cellular integrity and enhance early osteogenic activity, our findings position ferroptosis as a central regulatory node in bone cell biology. Importantly, the distinct mechanisms of Liproxstatin-1 and Coenzyme Q10 illustrate that ferroptosis suppression can be achieved through complementary molecular pathways, offering new opportunities for pathway-specific intervention. Beyond the immediate experimental outcomes, this work provides a conceptual framework for leveraging ferroptosis modulation in strategies aimed at lessening oxidative stress–related bone disorders and promoting regenerative processes. Our findings therefore contribute to a broader understanding of how redox-regulated cell death pathways influence skeletal homeostasis and identify potential therapeutic avenues for enhancing bone repair.

A potential future application of our insights into ferroptosis inhibition from this research could guide the design of next-generation biomaterials with superior bone-forming capabilities. Incorporating ferroptosis-inhibitory features into scaffolds or surface coatings could lead to the development of implants that better sustain stem cell survival and osteogenic function under oxidative stress. This strategy presents a compelling pathway for advancing regenerative medicine and optimizing bone repair and implant integration.

## Data Availability

The original contributions presented in this study are included in the article. Further inquiries can be directed to the corresponding authors.
